# People make mistakes: Obtaining accurate ground truth from continuous annotations of subjective constructs

**DOI:** 10.3758/s13428-024-02503-3

**Published:** 2024-09-30

**Authors:** Brandon M. Booth, Shrikanth S. Narayanan

**Affiliations:** 1 Department of Computer Science, University of Memphis, 38152 Memphis, TN USA; 2 Electrical and Computer Engineering Department, University of Southern California, 90089 Los Angeles, CA USA

**Keywords:** Continuous annotation, Validity, Reliability, Ordinal perception, Movie violence

## Abstract

Accurately representing changes in mental states over time is crucial for understanding their complex dynamics. However, there is little methodological research on the validity and reliability of human-produced continuous-time annotation of these states. We present a psychometric perspective on valid and reliable construct assessment, examine the robustness of interval-scale (e.g., values between zero and one) continuous-time annotation, and identify three major threats to validity and reliability in current approaches. We then propose a novel ground truth generation pipeline that combines emerging techniques for improving validity and robustness. We demonstrate its effectiveness in a case study involving crowd-sourced annotation of perceived violence in movies, where our pipeline achieves a .95 Spearman correlation in summarized ratings compared to a .15 baseline. These results suggest that highly accurate ground truth signals can be produced from continuous annotations using additional comparative annotation (e.g., a versus b) to correct structured errors, highlighting the need for a paradigm shift in robust construct measurement over time.

**Supplementary Information:**

The online version contains supplementary material available at 10.3758/s13428-024-02503-3.

## Introduction

Lord Kelvin famously stated, “if we cannot measure a thing, we cannot improve it” – a principle equally applicable to the study of human psychology as it is to the physical sciences. Understanding human mental states and experiences, or *constructs*, requires trustworthy measurements of their complex dynamicsare trustworthy. The effectiveness of intervention strategies aiming to improve human experiences, for example, ranging from extending learning opportunities by reengaging distracted students to calming anxious individuals when harmful stress is detected, relies on robust measurement of psychological states. Such systems can only serve their intended function if our quantitative measures of human constructs are valid and reliable.

By their very nature, subjective psychological constructs cannot be measured directly, and thus we rely on human assessment and annotation to obtain labels for perceived experiences. This work focuses on continuous annotation, where perceived construct valuations are efficiently produced by human observers (also called *annotators*) over time. For example, annotations comprised of interval-scale values (e.g., between zero and one) gathered while annotators view a stimulus may be used to rate student engagement levels (e.g., Booth et al. 2017) or to denote changes in dimensional emotion (i.e., valence and arousal) as they evolve over time (e.g., Kossaifi et al. 2019; Metallinou and Narayanan 2013; Mollahosseini et al. 2017; Ringeval et al. 2013; Sharma et al. 2019; Zafeiriou et al. 2017).

**Fig. 1:**
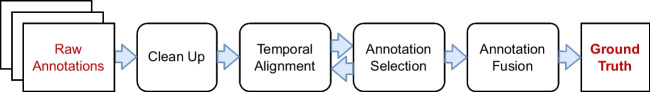
A general-purpose pipeline for ground truth generation from raw continuous annotations collected from human observers

Notably, for any given stimulus, multiple percepts of a construct may exist. In this work, we focus on the question of how to obtain a highly accurate representation of a construct’s dynamics in accordance with annotator consensus, which represents only the prevailing percept (though our methods should function for other percepts as well). Assuming annotations are collected independently from multiple annotators and a consensus set is identified, an impartial measure of a construct is typically formed by fusing them into a single continuous signal. This fused signal is then often used as the ground truth for modeling or machine learning.

Several methods have been proposed for fusing continuous human-produced annotations into a ground truth (e. g., Lopes et al. 2017; Mariooryad and Busso, 2015; Metallinou and Narayanan, 2013), though little research has assessed the validity of these ground truths. These methods often assume that human error during continuous annotation is unstructured with zero-mean noise, however, recent evidence shows that these errors are structured and pronounced (Booth et al., 2018b) and consistent across annotators (Booth & Narayanan, 2020a). Thus, there is a gap in foundational research on the validity of continuous annotation and also evidence threatening the validity of current approaches.

This work evaluates the validity and reliability of existing techniques used to generate ground truth from continuous annotations and proposes new techniques for improvement. We adopt notions of validity and reliability from psychometrics (a field which studies construct measurement) to assess the trustworthiness of these methods. Our proposed techniques leverage ordinal (comparative) human judgements to correct inaccuracies in continuous annotations. We demonstrate their effectiveness in a crowd-sourced experiment involving continuous annotation of movie violence intensity. The results confirm previous findings on continuous annotation errors and demonstrate that the errors can be mitigated using ordinal processing techniques. By embracing the structured mistakes that annotators make during continuous annotation (e.g., Booth et al. 2018b; Booth and Narayanan 2020a), we show that a carefully constructed ground truth based on ordinal comparisons and interpretations can improve validity and reliability over existing approaches.

## Background

### Continuous annotation for construct measurement

#### Definition

*Continuous annotation* is a measurement process whereby a human annotator provides a sequence of valuations for a construct over time. We focus on interval-scale (i.e., real-valued) valuations performed in real-time as the stimulus is perceived, hereafter called *continuous annotation* for simplicity. While some researchers employ this method to annotate multiple dimensions or constructs simultaneously (e.g., valence and arousal together Sharmaet al., 2019), we consider one-dimensional single construct continuous annotation in this work. Theseannotations may be collected in one shot as a stimulus is perceived or in segments that are later assembled to form a single annotation of the whole stimulus, similar to thin slicing (Ambady et al., 2000; Slepian et al., 2014).

#### Uses

Continuous annotations have been used to represent subjective human constructs such as dimensional affect (e.g., valence and arousal Abadi et al. 2015; Koelstra et al. 2012; Kossaifi et al. 2019; McKeown et al. 2011; Metallinou and Narayanan 2013; Ringeval et al. 2013; Soleymani et al. 2011), challenge/immersion (e.g., Beaudoin-Gagnon et al. 2019), and student engagement (e.g., Booth et al. 2017, 2018). These annotations help uncover latent information about the dynamics of human mental states and aid in modeling and understanding human perception of complex constructs.

### Establishing ground truth

To generate a ground truth representation of the majority percept of a construct, continuous annotations are collected from multiple annotators, inspected to find the subset forming a consensus, and then fused (e.g., via averaging) into a single time series signal. The purpose of fusing multiple annotations is to “average out” individual annotator’s biases and errors. Figure 1 illustrates the general processing stages that transform raw (i.e., unprocessed) continuous annotations into a ground-truth time series, explained below.

#### Raw annotations

Continuous annotations of a single stimulus are captured independently from multiple annotators. For example, annotators may be presented with an emotional video clip and asked to provide ratings of perceived emotional arousal in real-time as they observe it.

Various tools have been developed to facilitate this process and give annotators precise control over the ratings. Many data sets for emotional dynamics research use custom software with user interfaces (UIs) where the stimulus is presented in one window pane while ratings are collected in another (e.g., Booth et al. 2017; Cowie et al. 2000). Some tools enable annotators to finely adjust ratings using UI sliders controlled by a mouse or a keyboard (Booth et al., 2017; Cowie et al., 2000; Melhart et al., 2019), while others use specialized hardware devices like 3D motion controllers or joysticks (Lopes et al., 2017).

#### Data clean up

Errors in collecting raw annotations are corrected before further processing. For example, spurious missing values due to hardware failures may be imputed or filled with NaN (not-a-number) values, annotations may be resampled to a common rate (e.g., 10 Hz), or valuation errors from periods of annotator inattention may be adjusted or overwritten with NaN values to exclude them from subsequent ground truth computation. There are no standard tools for this stage, and the types of clean-up methods employed are often dependent on the needs of each study.

#### Temporal alignment

Continuous annotations may suffer from temporal misalignment due to differences in perceptuo-motor lag times or measurement instruments. Several methods and tools have been utilized for aligning the raw annotations. Mariooryad and Busso (2015) introduced a technique that shifts annotations uniformly in time (i.e., without temporal distortion) to maximize the pairwise mutual information between each pair of cleaned raw annotations. Dynamic time warping (DTW) is another common approach that corrects non-uniform time lags between pairs of raw annotations by finding the optimal matching between the samples in both time series that maximizes their alignment (Müller, 2007).

Several derivative methods of this DTW approach exist, such as canonical time warping (CTW) (Zhou & Torre, 2009), deep canonical time warping (Trigeorgis et al., 2016), and other generalized variants (Zhou & De la Torre, 2016, 2012), that introduce additional constraints to the optimization problem and solve it using different algorithms. While some methods achieve temporal alignment using only the cleaned annotations (e.g., DTW), others depend on features extracted from the stimulus (e.g., CTW using facial expressions or verbal/paraverbal features).

In practice, a uniform temporal shift method from Mariooryad and Busso (2015) has been shown to work well in controlled settings where little missing or invalid annotation data is present (Booth et al., 2018b; Mariooryad & Busso, 2015). DTW and its variants, however, may work well for annotations over larger periods of time when annotators are more likely to exhibit differing reaction lag times due to, for example, distractions, fatigue, or inattention (Booth et al., 2018a). Publicly available tools for this stage include a MATLAB package for uniform alignment (Mariooryad & Busso, 2015), a DTW library written in R (Giorgino et al., 2009), and a suite of tools for ground truth generation including time alignment methods (Stappen et al., 2021).

#### Annotation selection

In this stage, inlier annotations are selected to form a consensus and included in the subsequent annotation fusion stage while outliers are excluded. For well-controlled experimental settings, like laboratories, outliers from low-effort or distracted annotators are rare because adequate annotator attention can be ensured. In less controlled scenarios, like crowd-sourcing, where annotator attention cannot be monitored, outlier removal is necessary to exclude low-effort and inaccurate annotations from the ground truth. Pairwise correlation or multi-way agreement measures are often used to assess the relationship or correspondence among time-aligned annotations, dropping those that disagree with the majority or percepts of interest. Methods for assessing the relationship or agreement between annotations include Cronbach’s alpha (Aljanaki et al., 2017; Busso et al., 2008; McKeown et al., 2011; Metallinou & Narayanan, 2013), Krippendorff’s alpha (Yannakakis & Martinez, 2015), Pearson correlation (Kossaifi et al., 2017; Metallinou & Narayanan, 2013; Valstar et al., 2014; Zafeiriou et al., 2017; Ringeval et al., 2013), Cohen’s Kappa (Soleymani et al., 2011; Devillers et al., 2006; Ringeval et al., 2013), signed agreement (Nicolaou et al., 2010), and signed differential agreement (Booth & Narayanan, 2020a).

After selecting annotations using these strategies, it may be beneficial to realign only the selected annotations to maximize their mutual correspondence before fusion.

#### Annotation fusion

The cleaned and temporally aligned annotations are combined to form the ground truth time series. One simple technique is to average the annotations, as seen in previous studies (e.g., Mariooryad and Busso, 2013; Schuller et al. 2011). Alternatively, probabilistic methods such as Bayesian networks and Markov models treat the ground truth as a signal reconstruction problem, where each annotation is treated as a noisy representation of the true construct signal and the goal is to model and remove the noise. These methods propose different probabilistic noise models to characterize annotation errors (e.g., Gaussian noise) and use them to uncover the latent signal (i.e., the hidden true construct signal) for use as the ground truth (e.g., Gupta et al. 2016; Ramakrishna et al., 2016). Stappen et al. (2021) provide code implementing some fusion options.

Certain algorithms like CTW (Zhou & Torre, 2009) and dynamic probabilistic canonical correlation analysis (Nicolaou et al., 2014), aim to learn ground truth representations from both raw annotations and a set of features derived from the stimulus. For example, these features may include verbal and paraverbal aspects from audio (e.g., n-grams, pitch, jitter) or visual cues (e.g., facial expressions, body posture, background/foreground motion) from video. Fusion approaches like these may help construct a ground truth representation based on human perceptions aligned with the stimulus creators’ intent, such as the designed emotional arc of a movie. However, we urge caution when using these methods to understand human construct perception, as they impose additional constraints on the ground truth that may reduce construct validity. We elaborate on this point in Section Threats to reliability and validity.

### Assessing the quality of ground truth

An ideal ground truth accurately reflects the true dynamics of the construct it represents. In practice, constructs cannot be quantified directly, so we must rely on estimates of the trustworthiness of human-produced annotations and the continuous annotation process to assess the quality of the ground truth.

Psychometrics, a sub-field in psychology that studies measurement processes for constructs, offers an established perspective on evaluating the ground truth quality. The *Standards for Educational and Psychological Testing* (hereafter *Standards*) is a predominant authority providing three criteria for evaluating measurement quality: reliability, validity, and fairness (American Educational Research Association [AERA] et al., 2014). In the following subsections, we define reliability and validity and discuss them in the context of current research practices using continuous annotations. Although fairness is crucial, especially in high-stakes machine learning contexts (Booth et al., 2021), it is beyond the scope of this work.

#### Reliability assessment in prior studies

The *Standards* loosely defines the reliability of a measurement procedure as its ability to consistently produce the same results across multiple measurements. It is important because it establishes trust in the generalizability of a measurement process to novel stimuli. In the context of continuous annotations gathered from multiple annotators, the interrater reliability assesses the extent to which annotators can consistently distinguish between different stimuli. However, since continuous annotation is commonly used to rate subjective constructs, interrater reliability is difficult to measure since the true variations in the construct are unknown. Instead, interrater agreement measures are often used, which gauge the concordance of a collection of annotations gathered independently from various annotators.

Many research studies involving continuous annotation of subjective constructs report some measure of agreement to enhance confidence in the ground truth. Common agreement measures include concordance correlation coefficient, Kendal’s $\tau$, Cohen’s $\kappa$, Cronbach’s $\alpha$, Krippendorff’s $\alpha$, and intra-class correlation (e.g., Artstein and Poesio, 2008; Carletta, 1996; Koo and Li, 2016; Krippendorff, 2004; Reidsma and Carletta, 2008). For example, Metallinou and Narayanan (2013) report a Cronbach’s $\alpha > 0.7$ for emotional valence, arousal and dominance annotations, which corresponds to an “acceptable” level of agreement according to frequently cited (and somewhat controversial) measurement heuristics (DeVellis & Thorpe, 2021). Booth et al. (2017) report an intra-class correlation (ICC[3,k]) of 0.6 for student engagement annotations, which is a “good” level of agreement according to Cicchetti (1994). Sometimes correlations are used as agreement measures, such as Pearson or Spearman correlations, however, researchers caution against this since they are agnostic to translations in the rating scale (Booth & Narayanan, 2020a; Krippendorff, 2004; Ranganathan et al., 2017).

There are surprisingly few studies addressing issues related to the reliability of continuous annotation. Metallinou and Narayanan (2013) authored one of the first papers underscoring the need to extend reliability measures typically used for discrete signals to the time-continuous regime. The authors also argued that due to individual differences in construct valuation across annotators, the reliability among the annotations might best be assessed based on the relative differences, rather than absolute values, in annotations over time. This idea was further developed by Yannakakis et al. (2018) in an exposition proposing that perception of emotional dynamics is fundamentally ordinal. If true, then treating continuous annotations in a relative fashion and calculating reliability ordinally would be necessary. Yannakakis et al. (2018) further propose that annotators should be instructed to provide annotations directly in ordinal space (i.e., where changes over time are meaningful and scale values are meaningless), but that work does not offer a means to assess reliability. Booth et al. (2018b)have also independently observed and proposed that relative changes in continuous annotations are more meaningful than absolute scale interpretations. Booth and Narayanan (2020a) later demonstrated that interval-scale reliability metrics produce results misaligned with human intuition because of the ordinal manner in which people seem to perceive signals. Considering these insights, we discuss threats to the reliability of ground truths established from interval-scale interpretations later in Section Threats to reliability and validity.

#### Evidence of validity in prior studies

The *Standards* defines validity as the extent to which accumulated evidence supports a measurement process and its results (i.e., ground truth) for a specific purpose (e.g., machine learning for construct inference). *Standards* states that, “validity is ... the most fundamental consideration when developing tests and evaluating tests.” Essentially, without sufficient evidence of validity, the utility of the ground truth is questionable.

Few studies have investigated the validity of ground truth signals derived from continuous annotations, and most of these efforts address validity for a narrow range of constructs. In a study involving the annotation of emotional arousal, Li et al. (2015) provide some evidence of ground truth validity via its correspondence to arousal measures derived from galvanic skin response features. Additionally, Sharma et al. (2019) present a data set of 2D simultaneous continuous annotations of arousal and valence and some support for the validity of the annotations via comparison to annotators’ physiological features. The authors demonstrate that a low-dimensional distillation of the physiological features qualitatively aligns with interpretable clusters (e.g., scary, relaxing) of mean-summaries of each annotation. Though these analyses offer some evidence of validity, they rely upon the correspondence of physiological indicators to mental states like arousal and stress, which may have limitations outside of well-controlled contexts (Booth et al., 2022; DMello & Booth, 2022).

Some publications express concerns about the validity of interval-scale interpretations of continuous annotations. For example, Metallinou and Narayanan (2013) and Yannakakis et al. (2018) question the *cognitive process* validity (i.e., can annotators evaluate a construct in real time while observing a stimulus?) and *internal structure* validity (i.e., is the annotation structure consistent with the construct?) but do not provide any evidence for or against in either case. Other researchers (e.g., Booth et al., 2018b; Metallinou and Narayanan, 2013; Yannakakis et al., 2018) have suggested that the differential structure of interval-scale annotations may hold more relevant information about the dynamics of a construct than the annotated values themselves. This idea is further supported by Camilleri et al. (2017), which demonstrates that machine learning models have an easier time predicting relative changes (i.e., increases or decreases) in a construct compared to estimating values at any point in time. Though a numeric interpretation of continuous annotations has proven useful for predictive modeling, these works suggest the validity of an ordinal interpretation may be stronger.

To the best of our knowledge, Booth et al. (2018b) provide the only study directly examining the accuracy of continuous annotations. They conducted a pilot study where annotators rated the perceived dynamics of a known quantity (the intensity of a solid color) rather than a subjective construct so the accuracy of the continuous annotations could be measured directly. In two experiments, the study found that a simple time-aligned and frame-wise averaged ground truth achieved a correlation as low as $r=.906$. The authors proposed a novel error-correction algorithm based on an ordinal interpretation of the annotations, which substantially improved the correlation ($r=.967$)^1^. Though any ground truth correlation over $r=.9$ would generally be considered excellent, the authors provided evidence that the accuracy of ground truths derived from continuous annotations can be substantially improved when adopting ordinal interpretations of these annotations.

## Threats to reliability and validity

We discuss three major threats to the reliability and validity of ground truths derived from the general ground truth pipeline outlined in Fig. 1.

### Threat 1: systemic momentary annotation errors

Booth et al. (2018b) identify two common momentary annotation errors in a study comparing continuous human annotations to a known objective signal: 1) annotators overshoot intended values when marking increases or decreases, and 2) annotators may adjust values when no actual change in the stimulus has occurred. The authors hypothesize the second error occurs when annotators correct their values to better match their perception, even though the stimulus remains unchanged. Regardless of the underlying mechanisms causing these artifacts, they cannot be “averaged out” during fusion because they are systemic to many individual annotations.

### Threat 2: inconsistent valuation of constructs over time

If the values which annotators assign to the same stimuli over time are inconsistent with prior valuations, the validity of the ground truth may be compromised. Indeed, evidence suggests that annotators inconsistently rate the same or similar stimuli at disparate points in time, even within a single annotation task, despite accurately capturing changes (e.g., Booth et al. 2018b; Yannakakis et al. 2018; Metallinou and Narayanan, 2013; Camilleri et al. 2017), which may be due the ordinal nature of human perception (Yannakakis et al., 2018). Therefore, ground truths based on these annotations’ values over time, rather than their ordinal relationships (i.e., increases/decreases), will be less valid.

### Threat 3: annotation fusion using stimulus features

Methods that optimize ground truth representations based on both continuous annotations and features from the stimulus (e.g., facial expressions, paraverbal signals) risk producing invalid ground truths. These methods seek to improve the “learnability” of the construct by proposing a ground truth that aligns with both the continuous annotations and some combination of features (see Section Establishing ground truth for examples). However, if the features lack sufficient information to capture the construct dynamics, this approach can fail. These techniques invert the typical machine learning problem: they assume a specific relationship between the features and the construct, then derive a ground truth that fits the features. This can lead to ground truth representations that are easy for machines to learn and give a false impression that the trained learning models are able to predict the (human perceived) construct, but do not accurately reflect the actual construct. Consequently, this style of fusion threatens the reliability and validity of the ground truth, especially in domains where features cannot closely approximate the true construct dynamics (e.g., predicting stress from physiological features Booth et al., 2022).

### Novelty of current study

The remainder of this work describes and builds on a suite of methods individually designed to address one or more of these threats to validity and reliability when generating ground truth from continuous annotations based on the majority percept (Booth et al., 2018b; Booth & Narayanan, 2020a, 2019, 2020b). Together, these methods aim to correctsystemic momentary annotation errors (Threat 1), support and utilize ordinal interpretations of continuous annotations as valid and reliable information (Threat 2), and avoid relying on stimulus features or external information (Threat 3).

Previously, the methods were tested individually in small-scale controlled experiments. The present work combines these methods and evaluates their effectiveness when utilized together. We address two research questions: can the validity of the ground truth be improved when using all these methods together (RQ1), and do these techniques generate accurate ground truths from continuous annotations gathered in less controlled and more naturalistic settings (RQ2)?

To address these research questions, we propose a novel ground truth generation pipeline incorporating this suite of methods (i.e., Booth et al. 2018b; Booth and Narayanan, 2020a, 2019, 2020b) (RQ1). Then, we present a case study using crowd-sourced continuous annotations of perceived violence in movies to evaluate the pipeline’s effectiveness at generating a valid ground truth (RQ2). We focus on movie violence as a construct because it is subjective, yet content ratings authorities provide summarized ratings of movie violence, which we utilize to provide evidence of validity. Using crowd-sourced annotations highlights our approach’s effectiveness in a context where annotator agreement and attention/effort are especially challenging.

To summarize, the novel contributions in this work are:

1. Highlighting validity and reliability as crucial quality measures for ground truth representations and identifying threats to these in current continuous annotation practices.
2. Proposing a novel ground truth pipeline based on ordinal interpretations of continuous annotations to address these threats.
3. Introducing and sharing a dataset of crowd-sourced continuous annotations of movie violence in five Hollywood films and then evaluating the proposed ground truth generation pipeline with respect to a ground truth baseline.^2^

## Proposed ground truth pipeline

We present a pipeline for robust and valid ground truth generation which combines methods from our prior works that address the major threats identified earlier. This pipeline builds on the annotation fusion method from Booth et al. (2018b), which is designed to handle systemic annotation errors and inconsistencies by embracing an ordinal interpretation of annotators’ perceptions, thus addressing Threats 1 and 2 from Section Threats to reliability and validity, and relying only the annotations themselves to derive a ground truth, addressing Threat 3.

**Fig. 2:**
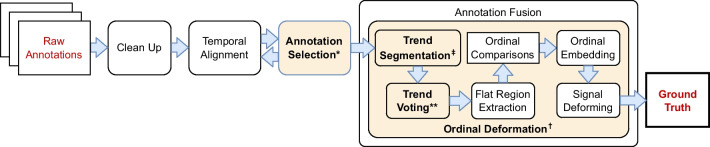
Proposed method for ground truth generation consistent with observations that annotators capture trends reliably and annotate values less reliably. Stages with a *shaded background* use methods from prior publications: $$^*$$Signed Differential Agreement Booth and Narayanan (2020a), $$^\dagger $$Perceptual Similarity Warping Framework Booth et al. (2018b), $$^{\ddagger }$$Trapezoidal Segmented Regression Booth and Narayanan (2019), $$^{**}$$Trapezoidal Segment Sequencing Booth and Narayanan (2020b)

Figure 2 illustrates the stages in our proposed pipeline, enhancing the basic annotation pipeline from Fig. 1. *Shaded boxes* indicate stages where specific methods from previous publications are applied to address these threats. Below, we provide an overview for this proposed pipeline and detail each stage.

### Overview

Figure 2 illustrates our proposed ground truth pipeline. First, raw annotations are cleaned and temporally aligned using domain-dependent best practices. In the annotation selection stage, the reliability of annotations is determined by examining agreement in trends rather than the values, and outlying annotations are excluded from further analysis. A method based primarily upon (Booth et al., 2018b) (which we call *Ordinal Deformation* here) forms the basis of the annotation fusion step where the selected and aligned annotations are corrected using ordinal comparisons and then fused to generate the ground truth.

The key innovation in the annotation fusion step is that the peaks, valleys, and plateaus in the annotation signals are treated as adjustable regions, modified based on human comparisons. Since these comparisons yield more reliable measures of similarity (as evidenced by Booth et al. 2018b; Booth and Narayanan 2020a and discussed below), the resulting ground truth values over time are internally consistent and the values can be compared across annotations (addressing Threat 2). The pipeline also avoids relying on stimulus features (addressing Threat 3) and uses ordinal comparisons to improve validity (addressing Threat 1).

**Fig. 3:**
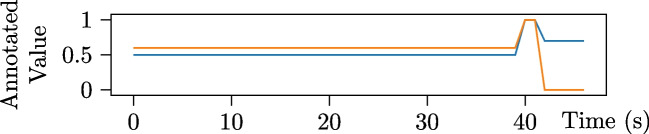
Hypothetical annotations of a stimulus, reproduced from Booth and Narayanan (2020a)

**Fig. 4:**
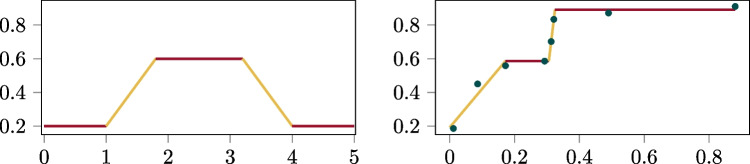
Two trapezoidal signal examples: A prototypical trapezoidal signal on the left, and the optimum four-segment trapezoidal signal fit to sample points on the right, reproduced from Booth and Narayanan (2019)

### Description of highlighted stages

Each shaded stage in Fig. 2 is detailed below. These stages are based on our observation in Booth et al. (2018b) that annotators reliably capture trends but struggle with assigning accurate values. Thus, each of the following stages assumes that only the ordinal information in each annotation is meaningful.

#### Annotation selection

This stage identifies the annotations that align sufficiently to form a majority consensus, filtering out unreliable annotations and alternative percepts. It involves two steps: measuring agreement and clustering annotations based on similarities or dissimilarities. The method used here is from our previous work (Booth & Narayanan, 2020a), where the agreement metric captures agreement in trends rather than values.

Per Booth and Narayanan (2020a), we derive an ordinal agreement metric based on two evidence-supported assumptions from Booth et al. (2018b):

1. **Construct perception is unique and approximately monotonic**:
  1 $$\begin{aligned} \frac{\text {d}P_i}{\text {d}z} \gtrapprox 0 \end{aligned}$$
2. Trends are reliably captured over time:
  2 $$\begin{aligned} \sum _t{\Big [\text {sgn}\Big (\frac{\text {d}T}{\text {d}t}\Big ) - \text {sgn}\Big (\frac{\text {d}}{\text {d}t}P_i[T(t)]\Big )\Big ]} \approx 0 \end{aligned}$$

Here, *T*(*t*) represents the true construct values over time, $P_i(z)$ is the unique and approximately monotonic perception function for annotator *i* for an observed percept with construct value *z* and, we denote the raw annotation from annotator *i* as $P_i[T(t)]$. In other words, Eq. 1 represents annotator i’s ability to perceive an increase in a construct as an increase and vice versa, while Eq. 2 captures annotator i’s ability to reliably perceive (and annotate) this over time. Using these formulations, we derive an equation for the similarity between two annotations:

3 $$\begin{aligned} \sum _t\Big [\text {sgn}\Big (P_i[T(t+\Delta )]-P_i[T(t)]\Big ) - \text {sgn}\Big (P_j[T(t+\Delta )]-P_j[T(t)]\Big )\Big ] \approx 0 \end{aligned}$$

where some small $\Delta >0$ is used to approximate a derivative and can be set to the sampling period (e.g., $\Delta =0.1$ seconds for a 10-Hz sampling rate) for discretely sampled signals like annotations.

In Booth and Narayanan (2020a), we derive an agreement measure called *signed differential agreement* (SDA) based on this similarity relationship. While commonly used measures report disagreement between the two simulated annotations in Fig. 3 (e.g., Kendall’s $\tau =-.38$, Cronbach’s $\alpha =-.18$, ICC=$-.15$, Krippendorff’s $\alpha =-.50$), SDA shows agreement (SDA$=1.0$) and is consistent with human opinion about these signals as well (Booth & Narayanan, 2020a). Despite the disagreement between these simulated annotations on the exact value, they identically agree on the trends, which is exactly what SDA measures.

Thus, we employ SDA from Booth and Narayanan (2020a) for measuring agreement as part of our proposed ground truth pipeline. SDA is formally defined as the normalized sum of sample-by-sample agreement between two signals of length *N* and has a range between [-1,1]:

4 $$\begin{aligned} \begin{array}{lll} \text {SDA} & = \frac{1}{N-1}\sum _{t=2}^N \delta [\text {sgn}(x_t-x_{t-1}),\text {sgn}(y_t-y_{t-1})]\\ \delta (p,q) & = {\left\{ \begin{array}{ll} 1 & p=q\\ -1 & p \ne q \end{array}\right. } \end{array} \end{aligned}$$

#### Ordinal deformation

This method combines annotations through multiple steps to create a robust and valid fusion. This fusion framework was first introduced in Booth et al. (2018b). Here we suggest modifications to certain steps (*Trend Segmentation* and *Trend Voting*) to ensure consistency with the principle that ordinal relationships should be trusted over annotated values. Details on each of the steps in this fusion stage are given below.

**Fig. 5:**
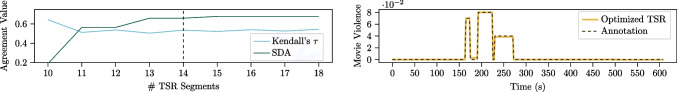
An example of our heuristic TSR optimization for a single annotation *i*. The *left figure* shows the two agreement measures considered in our heuristic for optimizing the number of TSR segments $T_i$, and the *dotted line* denotes the selected $T'_i$. The *right plot *shows the original annotation and the TSR approximation corresponding to $T'_i=14$ in this example

#### Trend segmentation

This initial step in our proposed fusion process partitions each annotation into segments where they primarily show an increase, decreasce, or remain relatively constant. In the original pipeline from Booth et al. (2018b), total variation denoising (TVD) was used for this, but TVD relies on potentially unreliable annotation values (Threats 1 and 2 from Section Threats to reliability and validity). Instead, we adopt trapezoidal segmented regression (TSR), introduced by Booth and Narayanan (2019), which addresses these concerns.

Trapezoidal segmented signals are similar to linear segmented signals in that they form a continuous function by connecting linear segments. The difference is that trapezoidal segmented signals require every other line segment to have zero slope while the segments between them have positive/negative slopes. This structure is a relaxation of the characteristic trapezoidal signal (see Fig. 4). Booth and Narayanan (2020b) demonstrate it is capable of approximating any one-dimensional continuous signal, such as an annotation, with arbitrary precision. The authors propose a dynamic program for optimally approximating any sampled continuous function given a budget of *T* segments. Furthermore, the authors show that increasing *T* beyond a certain point yields diminishing returns in minimizing approximation error, as long as it sufficiently captures the signal’s structure.

Selecting an initial *T* value was left as an open question in Booth and Narayanan (2019). Balancing two conflicting goals poses a challenge: *T* should be large enough to minimize the TSR regression error yet as small as possible to reduce the complexity of the regression and minimize the amount of additional ordinal comparisons needed downstream in the pipeline. Thus, selecting the optimal *T* value is a pareto-optimal problem with no single “best” solution.

In this work, we propose a heuristic search to help balance these two goals. First, an initial parameter for the number of segments, $\hat{T}_i$, is approximated via human inspection for each annotation *i* by counting the number of peaks, valleys, plateaus, and trends. Next, for each annotation *i*, candidate TSRs are computed for segment counts $T_i \in \mathbb {T}_i = \{\lfloor \frac{4}{5}\hat{T}_i \rfloor , \lfloor \frac{4}{5}\hat{T}_i \rfloor +1, ..., \lceil \frac{6}{5}\hat{T}_i \rceil \}$. To select a Pareto-optimal $T'_i$ from this range, we introduce a heuristic defined over two agreement measures, SDA and Kendall’s $\tau$, which are used to compute an agreement score between each TSR and the annotation it approximates. $T'_i$ is chosen by finding the $T_i$ corresponding to the first local maximum SDA value starting from $\min \mathbb {T}_i$ and stepping upwards, then finding $T'_i$ corresponding to the next local maximum Kendall’s $\tau$ value starting from that $T_i$ and stepping upwards to $\max \mathbb {T}_i$.

Figure 5 (left) shows an example of this heuristic optimization, with the resulting TSR approximation shown on the right for a sample annotation from the case study detailed in Section Case study. In this and most cases, locally maximizing SDA would have been sufficient, but we discovered a few cases where this led to poor TSR approximations stemming from an underestimated initial $\hat{T}_i$ from human inspection. Adding Kendall’s $\tau$ as a second step introduces a “fail safe” that minimizes the risk of underestimating $T'_i$ (which would lead to worse regression error) while avoiding excessively large values. Although many other optimization strategies with different heuristics are possible, as noted in Booth and Narayanan (2019), once *T* is large enough to capture the structure of an annotation, its agreement with the original annotation becomes substantially less sensitive to further increases in *T*. There is room for improvement in this step, but the results in Section Results show that this simple heuristic performs well.

#### Trend voting

Once the annotations are individually approximated using TSR, where the trends (i.e., increasing, decreasing, or constant) can easily be identified, we utilize a voting mechanism to reach a consensus about the construct variation on a sample-by-sample basis. Booth and Narayanan (2020b) proposed a trend-based voting strategy derived from these TSR approximations for each annotation, which we employ here. Samples occurring within line segments with a zero slope in the TSR for each annotation receive a value of zero, samples with positive-sloped line segments receive a +1, and samples with negative-sloped line segments receive a -1. Thus each annotation *i* is transformed into a sequence of values in $\{-1,0,1\}$ called a trapezoidal segment sequence (TSS), and then majority voting across these sequences for each annotation yields a merged TSS.

#### Flat region extraction

This step uses the merged TSS to extract intervals of time where the construct remains constant. The TSS representation simplifies this process by allowing us to extract the contiguous subsequences of TSS samples containing all zeros. For each window in time corresponding to these contiguous zero values, an excerpt (e.g., a movie clip) of the stimulus is extracted, forming a collection of excerpts where the construct is approximately constant.

#### Ordinal comparisons

In this step, additional annotations are collected as ordinal comparisons to help sort the stimulus excerpts by the construct of interest. In Booth et al. (2018b), triplet comparisons are employed as a general-purpose approach for assessing excerpt similarity and ordering excerpts where annotators may have difficulty determining their ordinal relationship (e.g., for difficult constructs like “silliness”). For constructs where annotators can easily make ordinal comparisons (e.g., valence, arousal), pairwise comparisons can be used as they provide more information (i.e., in an information-theoretic sense; Jain et al., 2016) than triplet comparisons for ordinal embedding. We use pairwise comparisons in the Case study Section.

#### Ordinal embedding

This step assigns values to each stimulus excerpt, where the construct value remains approximately constant, so that the resulting ground truth is accurate and consistent (Threats 1 and 2). Ordinal embedding problems attempt to learn a (typically) lower dimension embedding that preserves a similarity relationship between subsets of data points. In Booth et al. (2018b), this is formulated for triplets and solved using a t-stochastic triplet embedding (t-STE) solver (Van Der Maaten & Weinberger, 2012). Though other solvers have been proposed, we employ t-STE because it effectively groups similar points and separates dissimilar ones, leading to simpler solutions, as noted in several works (Booth et al., 2018b; Mundnich et al., 2019; Van Der Maaten & Weinberger, 2012).

**Fig. 6:**
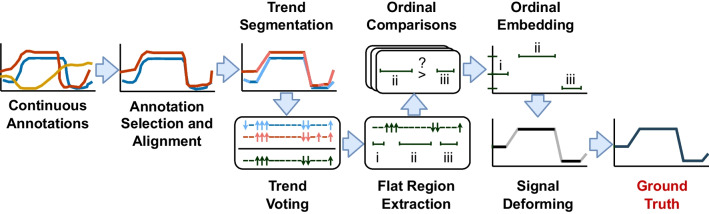
An illustration of the proposed pipeline at each stage for sample continuous annotations

The triplet embedding problem is formulated as follows: given a set of inputs $\mathcal {Y} = \{y_1,...,y_n\}$ with each $y \in \mathbb {R}^m$ and a set of similarity relations on 3-tuples from $\mathcal {Y}$ of the form $s(y_i,y_j) < s(y_i,y_k)$ where $\{i,j,k\}$ is a 3-subset of $\{1,2,...,n\}$, the goal is to find a set $\mathcal {Z} = \{z_1,...,z_n\}$ with each $z \in \mathbb {R}^d$ such that:

$$\begin{aligned} \Vert z_i-z_j\Vert< \Vert z_i-z_k\Vert \Longleftrightarrow s(y_i,y_j) < s(y_i,y_k) \end{aligned}$$

for some norm defined over $\mathbb {R}^d$ (where $d=1$ corresponds to a single construct dimension). These triplet comparisons express a similarity relationship where sample *i* is more similar to sample *j* than *k*. Collecting comparisons from humans over triplets has been studied and proven useful in other works (e.g., Jain et al. 2016, Van Der Maaten and Weinberger 2012).

For our application, we focus on pairwise ordinal comparisons but demonstrate the flexibility of the t-STE solver by converting pairwise comparisons into triplets. To do so, we need a common reference for all comparisons to serve as the third item in each triplet, thus we create a hypothetical dummy excerpt *r* to serve as the lowest ranked excerpt (i.e., having the smallest possible construct value such that $y_r < y_i$ for all *i*). Then, for each pair $(y_j, y_k)$ where an annotator determines that $y_j < y_k$ for the construct, we generate the corresponding triplet $\{y_r, y_j, y_k\}$ such that $s(y_r, y_j) < s(y_r, y_k)$. In other words, if excerpt *j* ranks lower in construct value according to the pairwise comparison, then the corresponding triplet indicates it is more similar to the smallest excerpt *r* than excerpt *k*. The beauty of this approach is that no additional comparisons are needed since $y_r$ is always assumed to be smaller than all other $y_i$. Once the embedding is generated from these triplets, the points in $\mathcal {Z}$ may need to be reversed to ensure the point $z_r$ corresponding to the dummy reference $y_r$ has the smallest value rather than the largest value. This is necessary because triplets only capture similarities while pairwise comparisons capture ordinal relationships, so some information about the proper orientation of the embedding within the construct scale is lost. Once the embedding is reoriented as needed, $z_r$ can safely be discarded.

#### Signal deforming

In the original implementation, Booth et al. (2018b) proposed constructing a ground truth from the selected and aligned annotations and the ordinal embedding results by utilizing a piecewise linear deformation function. In this work, we propose a simpler approach where the ground truth is reconstructed directly from: 1) the windows of time where the construct remains approximately constant (taken from the merged TSS from the *Trend Voting* step), and 2) the ordinal embedding results.

First, within each time window with an approximately constant construct value (identified in the *Flat Region Extraction* stage), we set the ground truth to a constant function whose value comes from the corresponding excerpt’s value in the ordinal embedding. Then the samples between these constant segments are connected using piecewise linear interpolation, resulting in a trapezoidal signal. This approach corrects both momentary valuation errors and long-term valuation inconsistencies (addressing Threats 1 and 2) without using any information or features from the stimulus itself (addressing Threat 3).

### Proposed pipeline example

Figure 6 illustrates the effect of the proposed pipeline from Fig. 2 on sample annotations. For simplicity, the clean-up, temporal alignment, and annotation selection stages are combined.

## Case study

We present a case study involving the continuous annotation of movie violence by a panel of crowd-sourced annotators. Perception of violence in movies is inherently subjective, with individual opinions varying on the intensity and nature of violent events. However, cultural norms shape these perceptions in an intersubjective manner, leading to a shared interpretation of the construct. This notion is supported by the existence of movie rating authorities (e.g., Motion Picture Association of America, Common Sense Media) that aim to provide ratings of movie violence reflective of public attitudes towards violence and thus enable viewers to make informed decisions.

We believe that intersubjective constructs with established rating authorities provide an ideal context for assessing the validity of majority consensus ground truths derived from continuous annotations since the authorities’ ratings serve as a benchmark for comparison. In this section, we describe the data set and continuous annotation collection procedures, which we later use to evaluate the robustness of the proposed ground truth technique.

### Data set description

We collected continuous annotations of movie violence in real-time from annotators recruited from Amazon’s Mechanical Turk, a crowd-sourcing platform. The movies, annotation protocol, and ground truth generation methods are described below.

#### Movie description and violence ratings

Five Hollywood films from the top grossing list between 2018-2019 were selected based on two criteria: violence rating and total running time. Violence ratings were obtained from Common Sense Media (CSM), which reviews and rates major Hollywood films on a discrete scale from 1 to 5. From each of these five violence categories, the shortest full-length feature film was chosen to minimize annotation costs. In ascending order of violence ratings, these movies were: *The Hustle* (2019), *Good Boys* (2019), *The Peanut Butter Falcon* (2019), *The Possession of Hannah Grace* (2018), *Rambo: Last Blood* (2019).

Each movie was divided into approximately 10-min segments to promote annotator attentiveness during the annotation process. The boundary for each cut was determined manually such that it aligned with the scene transition closest to the end of the 10-minute period. This ensured scenes were fully contained within one clip and helped mitigate the risk of scene-relevant context being separated across clips. Advertisements, previews, title screens, or non-narrative segments at the beginning or the end of the films were trimmed before segmentation. The cut times used to partition each film into clips are listed in Table S1 in the Supplementary Materials.

#### Annotator recruitment and annotation protocol

Annotations of perceived violence were obtained from volunteer workers on Amazon’s Mechanical Turk. Participants were presented with a task description and a warning about the potential amount of violence in the clips (see Fig. S1). They had the option to decline the annotation task. Those who chose to proceed were directed to a second page explaining the continuous annotation task and user interface (see Fig. S2).

After reading the instructions, participants were given an opportunity to practice using the interface by annotating a short video clip, which was not recorded. This step could be repeated as many times as needed until annotators felt comfortable with the interface. Once ready, a random video clip needing annotation was loaded, and annotation began when they pressed any button. Each clip was annotated by 10 different participants, who were compensated $1 USD per movie clip annotation task.

The PAGAN annotation software was used to collect continuous-scale annotations from participants in real-time as they viewed a movie clip (Melhart et al., 2019). We implemented a bounded interval-scale annotation format within PAGAN, preventing annotations from exceeding the minimum and maximum boundaries (see Fig. S2). This resulted in decimal-valued annotations recorded as time series data with values ranging from -100 to 100, though the scale numbers were hidden from annotators within the PAGAN interface. To adjust the values, annotators used arrow keys to move the label trace up or down along the construct scale within these bounds over time. PAGAN captured values as often as it was able (usually between 10–100 ms) and only logged value changes.

#### Inclusion criteria

All annotations were included despite varying quality due to missing data, disagreements, and possible annotator inattention. There were two reasons for this. First, perceptions of violence vary across individuals and across cultures, so excluding low-effort or adversarial responses may incidentally remove genuine annotations. Our goal was to demonstrate the robustness of the proposed method for obtaining an accurate majority percept ground truth in spite of these potentially varied perspectives. Second, the subsequent *Annotation Selection* stage (refer to Fig. 2) already filters out low-quality annotations, so we avoid redundant inclusion/exclusion processing here.

**Fig. 7:**
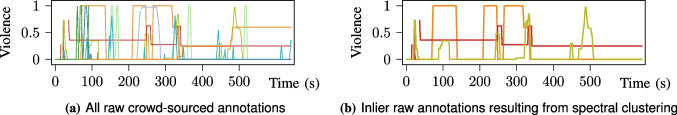
Raw crowd-sourced annotations of perceived violence over time in cut 4 from *The Hustle* (*left*) and the raw, unaligned, *inlier* annotations from the *Annotation Selection* stage (right)

### Ground truth generation

We generated two ground truths, one using the proposed pipeline and one baseline approach using a sample-wise average that performed well in Booth et al. (2018b). For a fair comparison, both methods used the same data cleanup, temporal alignment, and annotation selection strategies. Details on the implementation of these stages in our case study are given below.

#### Data clean up

To facilitate alignment and reduce overall complexity, all annotations were resampled to 1 Hz using linear interpolation. We found a rate of one sample per second to be more than fast enough to capture the highest frequency changes. Missing data in the annotations, corresponding to lapses in time with different annotated values on either end and no samples in between,) were flagged using not-a-number (NaN) values in the resampled version.

#### Temporal alignment and annotation selection

Since our focus is on capturing the majority consensus, we treated annotation selection as a binary clustering problem. Spectral clustering consistently achieves top performance in general types of clustering problems, so we used it to bifurcate the annotations based on the similarities between each pair of annotations (Rodriguez et al., 2019).

To form the similarity matrix required for spectral clustering, we first time-aligned each unique annotation pair using DTW, then we used SDA to measure pairwise differential agreement. As noted in Section Establishing ground truth, DTW is effective when there are variations in time delays between annotations, which was relevant in our study due to annotator lag and transmission delays. DTW requires a reference signal for alignment, so without loss of generality, we randomly chose one annotation from each pair as the reference and then aligned the two using DTW with a symmetric Sakoe-Chiba step pattern constraining the maximum temporal distortion to five seconds (Müller, 2007). Then, we used SDA as our agreement measure because it is agnostic to individual differences in valuation and is consistent with observations about the ordinal nature of perception. We measured SDA between each pair of aligned annotations, ignoring samples containing NaN values, and then populated an affinity matrix for two-class spectral clustering using these SDA similarities. The cluster with the highest average SDA value for each clip was selected as the inlier group, while the other annotations were excluded from further analysis. Table S2 shows the number of annotations selected as inliers for each movie clip.

#### Inlier temporal alignment

To further compensate for variance in human perception time and input lag, all inlier annotations for each clip were temporally aligned with respect to each other. As before, DTW requires a reference signal for alignment, and some works have proposed using features extracted from the stimulus as this reference (e.g., Nicolaou et al. 2014; Booth et al. 2018a). However, this approach presumes a correspondence between the selected (or crafted) feature(s) and the subjective label (i.e., violence), which can threaten the validity of ground truth as discussed in Section Assessing the quality of ground truth. Instead, we leveraged the agreement between each pair of annotations from the previous step and then selected the one with the largest average agreement to serve as the reference. The remaining inlier annotations were aligned to this reference using DTW again with a symmetric Sakoe-Chiba step pattern constraining the maximum temporal distortion to five seconds (Müller, 2007).

As a side note, we also attempted to use the alignment technique proposed by Mariooryad and Busso (2015), which applies per-annotation temporal correction based on mutual information and has been effective in other controlled studies (Booth et al., 2018b). However, in this crowd-sourced annotation study, this alignment method was unable to find a temporal shift that fit within our generous five-second maximum for the selected annotations. We hypothesize this may be due to unique character of the continuous annotations in this case study, which feature many long periods without perceived changes in violence (see Fig. 7a).

#### Annotation fusion

We separately employed two fusion methods to generate the baseline and proposed ground truths.

*Baseline:* The selected and temporally aligned annotations were averaged sample-wise for each movie clip. The fused annotations for each clip were then stitched together in sequence to form a single ground truth signal for the entire movie.

*Proposed method:* We applied the *Trend Segmentation*, *Trend Voting*, and *Flat Region Extraction* stages as described in Section Proposed ground truth pipeline to produce a set of movie clip excerpts where the perceived violence levels remained constant. Table S3 lists the number of flat regions extracted from each movie clip, corresponding to excerpts where the level of violence did not change according to the annotations. For the *Ordinal Comparison* stage, we recruited a separate set of crowd-sourced annotators from Amazon’s Mechanical Turk. These annotators were given instructions (Fig. S3), shown two movie clip excerpts, and then asked to select the less violent of the two.

Each presented pair of movie clip excerpts was randomly selected from all possible pairs while avoiding duplication. To help minimize total annotation costs, we collected these pairwise comparative annotations in increments of 5000 at a time, after which we used all annotations gathered thus far to generate a candidate ground truth signal. We repeated this process until the resulting signal did not significantly differ from the previous one, measured by Spearman correlation. We chose a stopping threshold of 0.8, corresponding to a “very strong” correlation according to Chan (2003), and we stopped after collecting 20,000 unique comparisons (out of a total possible unique 53,301 pairs) since the candidate ground truths’ Spearman correlations with the previous ones exceeded 0.8 for all movies (average $\rho =0.91$). Table S4 lists these correlations for each movie, computed after each batch of 5000 comparative annotations.

### Evaluation of candidate ground truths

We evaluated the baseline and proposed ground truth signals by comparing them to the CSM movie violence ratings. To achieve this, a many-to-one function was applied to reduce them to scalar values. Intuitively, movie violence ratings are produced with the intention of providing information about the peak (i.e., maximum) levels of violence in films, but it is unclear whether CSM summarizes violence throughout a movie in this way. Therefore, we tested several aggregation functions for each of the proposed ground truths: *min*, *max*, *mean*, *median*, and *sum*. Finally, we employed Spearman correlation to compare the aggregated candidate ground truths against the CSM violence ratings.

## Results

Figure 7a shows a representative sample of the raw annotations collected in this experiment. Each plotted line represents the annotation trace for a single annotator, and Fig. 7b shows the three lines selected as inliers in the *Annotation Selection* stage (note the annotations are not temporally aligned in this figure). Tables S2-S4 provide interim results about the number of annotations selected per movie clip, the number of excerpts extracted per clip by the *Flat Region Extraction* step, and the similarity between ground truth candidates after each batch of 5000 pairwise annotations. Table S5 also lists several agreement measures for the inlier and temporally aligned annotations for each movie clip.

**Fig. 8:**
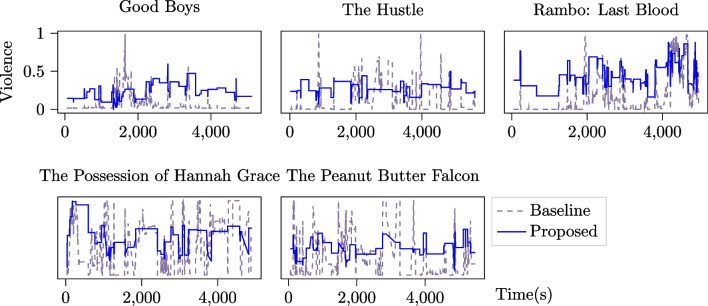
Plots of the ground truth for the baseline and proposed methods. Note that due to the monotonic and translational invariance of ordinal embeddings, the values in the proposed ground truth cannot be compared directly to the values in the baseline, but the values are comparable across movies within the same ground truth method

Figure 8 plots the baseline ground truths (dotted lines) and the proposed ground truths (solid lines) for each movie. The proposed ground truths have been uniformly scaled into the [0,1] range for visualization purposes, but the violence values between the baseline and proposed signals cannot be compared directly. This is because values resulting from ordinal embeddings in the final fusion stage are only consistent across these annotations and not anchored to a specific construct scale.^3^

**Fig. 9:**
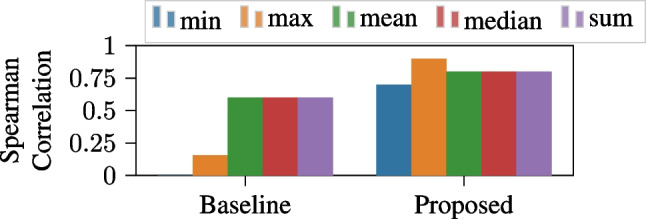
Spearman correlation between the CSM ratings and various aggregations of the baseline and proposed ground truths

Finally, Fig. 9 shows the Spearman correlation between the CSM violence ratings for each movie and the values obtained by applying different aggregation functions on both the baseline and proposed ground truth signals.

## Discussion

### Main findings

The results in Fig. 9 show that the proposed ground truth pipeline achieves a higher correlation with the CSM ratings than the baseline ground truth method, somewhat surprisingly, *regardless of the aggregation function used*. This suggests that the proposed ground truth more accurately represents the perceived dynamics of movie violence ratings.

Assuming the *max* aggregation function correctly summarizes continuous movie violence ratings, the Spearman correlation for the proposed method ($\rho =.95$) is substantially better than the baseline method ($\rho =.15$). Figure 8 reveals that annotators tended to rate violence levels as high in every film (i.e., close to a rating of 1.0), regardless of its CSM violence rating. This behavior occurred even though the annotators were explicitly asked to use the upper boundary of the scale for “extreme” levels of violence. This phenomenon may in part be explained by the lack of annotator training in our study protocol, which may have helped to mitigate this effect.

However, Fig. 8 also shows that the baseline often exaggerates the rate of change of perceived violence, in particular for *The Possession of Hannah Grace* and *The Peanut Butter Falcon*, where it appears to oscillate between minimum and maximum levels of violence throughout each film. These findings corroborate prior observations (e.g., from; Booth et al. 2018b; Metallinou and Narayanan 2013; Yannakakis et al. 2018) about overshooting effects (Threat 1) and inconsistent valuations of violence at different points in time (Threat 2).

The steps in our proposed pipeline are designed to enhance reliability and validity by addressing the three major threats discussed in Section Threats to reliability and validity. In our proposed approach, the *Annotation Selection* and *Annotation Fusion* stages utilize techniques based on ordinal information in the continuous annotations, therefore, as long as the annotations properly capture a construct’s increases/decreases, our proposed ground truth is agnostic to annotation value errors (Threat 1). Further, our use of ordinal comparisons and ordinal embedding in the *Annotation Fusion* stage helps correct inconsistencies in the valuation of movie clips with similar violence levels occurring at different points in time (Threat 2). Finally, since no external information (e.g., audio or video features) is used at any stage, our proposed method avoids contaminating the ground truth with irrelevant data, ensuring it accurately reflects the perceived construct dynamics according to the annotations (Threat 3). Hence, our proposed ground truth generation methodology is more reliable and more valid than the baseline, as it leverages the improved reliability of ordinal interpretations of continuous annotations (Yannakakis et al., 2018; Booth et al., 2018b) and uses ordinal comparisons to correct valuation errors.

In summary, achieving robust continuous ground truth is complex due to reliability and validity threats. We have demonstrated one possible approach for improving the quality of continuous ground truths of subjective constructs, primarily utilizing ordinal methods, but more work is needed to test the replicability this approach. We hope that our discussion of validity and reliability and the proposed ground truth pipeline will serve as a foundation for future efforts examining the quality of continuous measures of human constructs.

### Limitations and future work

While many of the methods used in our proposed pipeline have prior evidence supporting their validity, the additional evidence in this work is limited to one specific construct from five movies. Future work is needed to validate fully ordinal approaches to generating ground truth from continuous annotations. Furthermore, generating a stable ordinal embedding required over 20, 000 pairwise comparisons to generate a stable ordinal embedding. Although these comparisons are essential for maximizing the validity of the resulting ground truth, the cost and time required to collect them may be prohibitive at larger scales. More methodological work is necessary to minimize the number of comparisons needed to achieve similar results.

Additionally, to maximize their utility, the ground truths produced by our proposed approach need to be comparable to those produced by other means. Presently, these values cannot be directly compared to other ground truth signals due to the translational and monotonic invariance of ordinal embeddings. This issue could be remedied by including reference stimuli with known construct valuations (i.e., *anchors*) in the *Ordinal Comparisons* step. This anchoring technique has been successfully used in Likert-scale assessments for summative construct measurement (e.g., Crane et al. 2016), and future work should assess its effectiveness in the continuous domain.

Lastly, while our work touches on ordinal cognition and demonstrates that a comparative, ordinal approach to interpreting annotations can improve accuracy, the contributions in this work serve as a starting point for further exploration of the approach’s validity. Discussions of validity are usually divided into distinct types, such as content validity (are all dimensions of the construct considered?), concurrent validity (how does the measure compare to established measures?), and construct validity (does the measure assess the intended construct?). There are different perspectives on how types of validity evidence should be defined and interpreted, but regardless of these choices, the Standards states, “[validity] is the degree to which all the accumulated evidence supports the intended interpretation of [annotations] for the proposed use” (AERA et al., 2014, p.14). This work provides some empirical validity evidence based on the correspondence of summarized annotations to established measures (a type of concurrent validity) and explains how ordinal interpretations of annotations better align with perceived construct variations (evidence for internal structure and response process validity; AERA et al., 2014. However, other questions remain, such as whether this ground truth approach works for other constructs, multi-dimensional annotations (e.g., affect via valence and arousal), or multiple percepts. Further, though evidence from Booth et al. (2017) suggests the dynamics of the ground truth can be trusted provided that the annotations are reliable, we only evaluated the validity of the summarized ground truth (e.g., via min or max), so future work should aim to validate the ground truth dynamics in a similar context. In particular, the agreement between the continuous annotations for some movie clips was low or even negative (see Table S5), suggesting there was no consensus among the 10 crowd-sourced annotations per clip about movie violence dynamics. Future work should first acquire sufficiently reliable annotations of the consensus percept for all stimuli and then evaluate the validity of the dynamics of the resulting ground truth. Finally, the reliability and validity of the ground truth have only been evaluated for interval-scale continuous-time annotations. Future research should evaluate the validity of this approach using other continuous-time variants (e.g., ordinal continuous annotations Lopes et al., 2017) and seek further evidence for (or against) validity and reliability.

## Conclusion

Accurate modeling of human construct dynamics relies on robust ground truths derived from continuous annotations and on the validity and reliability of the methods used to generate them. Current techniques for measuring constructs continuously struggle with systemic annotation errors, potential contamination from external sources (i.e., stimulus features), and inconsistencies in the valuation of a construct over time.

This study demonstrates that the validity of continuous ground truth signals can be improved when reliable and trustworthy procedures, based on ordinal interpretations of continuous annotations, are used at each stage. These procedures differ greatly from those commonly used in research and practice, suggesting a need for a shift in best practices and emphasizing the importance of future validation and replication studies for valid and reliable continuous ground truth generation.

## Open practices statement

The code, experiments, and results used to support the findings of this study are available at https://doi.org/10.5281/zenodo.8085249 under the Creative Commons Attribution-NonCommercial-ShareAlike 4.0 International license (CC BY-NC-SA 4.0). None of the experiments were preregistered.

## Supplementary materials

Figures S1-S3 provide images of the information participants were given prior to and during their participation in the case study. Tables S1-S5 provide metadata and information about the quantity and quality of the data collected during the case study.

## Supplementary Information

Below is the link to the electronic supplementary material.

Supplementary file 1 (pdf 157 KB)

## Data Availability

The dataset and results are available at https://doi.org/10.5281/zenodo.8085249

## Footnotes

1 This explains an additional 11% of the variance using the $r^2$ approximation for explained variance.

2 Code and results for the dataset and experiments are available at https://doi.org/10.5281/zenodo.8085249

3 This could be remedied by including clips with known violence levels in the ordinal embedding (i.e., *anchors*).

## Code Availability

Code implementing the methods and producing the results are available at the same link above.
