# Supplementary material for: People make mistakes: Obtaining accurate ground truth from continuous annotations of subjective constructs
Source: Behav Res Methods. 2024 Sep 30;56(8):8784–800. doi: 10.3758/s13428-024-02503-3 (PMC11525321; doi:10.3758/s13428-024-02503-3)
Supplement: Supplementary file 1 — (pdf 157 KB) [file 13428_2024_2503_MOESM1_ESM.pdf]

Appendix

Supplemental Materials

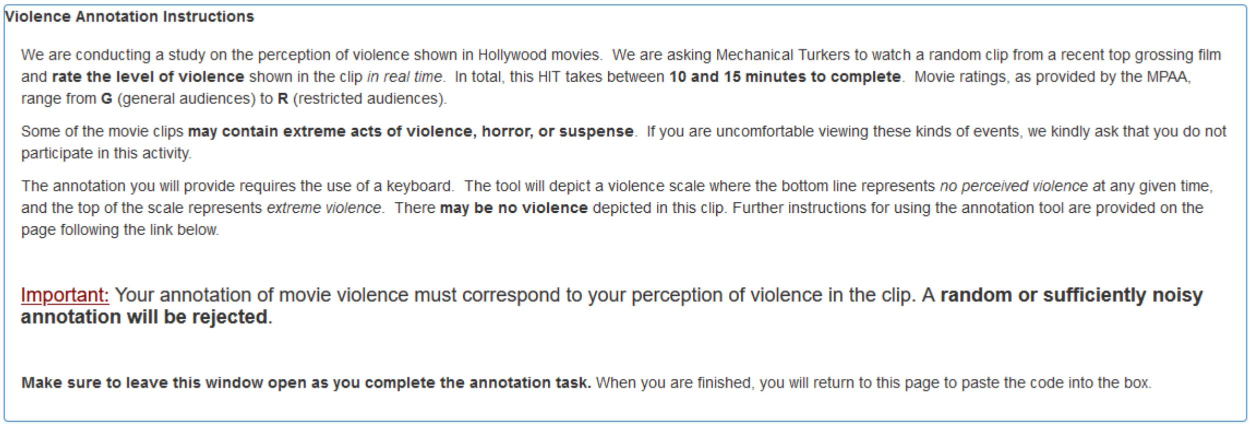

Violence Annotation Instructions

We are conducting a study on the perception of violence shown in Hollywood movies. We are asking Mechanical Turkers to watch a random clip from a recent top grossing film and **rate the level of violence** shown in the clip *in real time*. In total, this HIT takes between **10 and 15 minutes to complete**. Movie ratings, as provided by the MPAA, range from **G** (general audiences) to **R** (restricted audiences).

Some of the movie clips **may contain extreme acts of violence, horror, or suspense**. If you are uncomfortable viewing these kinds of events, we kindly ask that you do not participate in this activity.

The annotation you will provide requires the use of a keyboard. The tool will depict a violence scale where the bottom line represents *no perceived violence* at any given time, and the top of the scale represents *extreme violence*. There **may be no violence** depicted in this clip. Further instructions for using the annotation tool are provided on the page following the link below.

Important: Your annotation of movie violence must correspond to your perception of violence in the clip. A **random or sufficiently noisy annotation will be rejected**.

Make sure to leave this window open as you complete the annotation task. When you are finished, you will return to this page to paste the code into the box.

Figure S1: Landing page for Mechanical Turk workers interested in participating in our continuous annotation experiment.

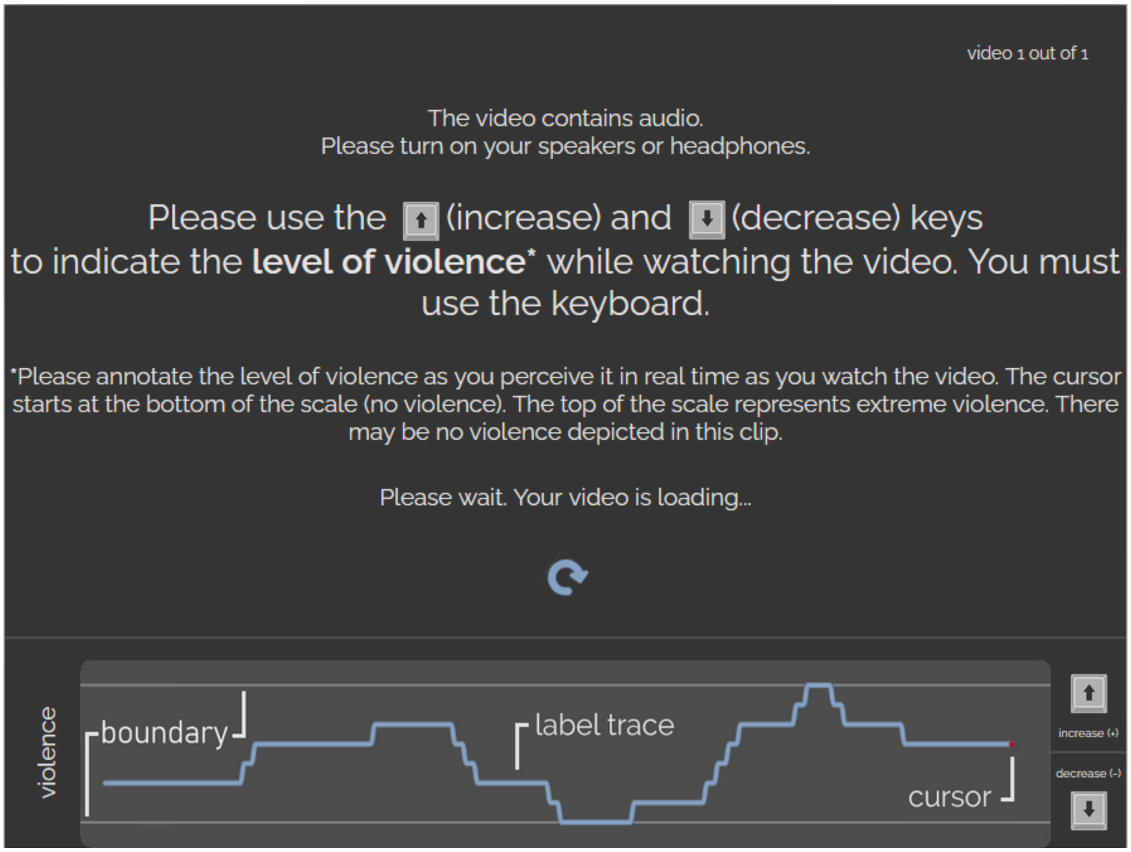

Figure S2: Landing page for Mechanical Turk workers electing to participate in our experiment involving continuous annotation of movie violence.

> *Two video clips of different lengths and from various Hollywood films are displayed: video A, video B. View the entirety of both clips, one at a time, then select the one that portrays the least amount of violence.*
>
> *Though some of the clips may be taken from the same movie, or though you may even be familiar with the films, you should focus only on the amount of violence displayed within in each clip. Some clips may be extremely short.*

Figure S3: Instructions presented to the second set of annotators before comparing perceived violence in movie clip excerpts.

**Movie Name**	Start	C1	C2	C3	C4	C5	C6	C7	C8	C9	C10	C11
The Hustle	15	537	1247	1844	2486	3106	3630	4177	4872	5332	5521	5612
Good Boys	54	598	1281	1886	2588	3121	3729	4405	5121			
The Peanut Butter Falcon	53	620	1255	1964	2554	3264	3842	4548	5116	5469		
The Possession of Hannah Grace	34	596	1192	1795	2414	3014	3713	4261	4901			
Rambo: Last Blood	51	659	1265	1884	2468	3008	3608	4206	4773	4959		

All times are listed in seconds. Start = start time for the first clip (excluding the movie title), C1 = end time for clip 1 and start time for clip 2, etc.

Table S1: Movie clip cut times for the five selected films.

Movie Name	Movie Clips										
	C1	C2	C3	C4	C5	C6	C7	C8	C9	C10	C11
The Hustle	4	3	3	3	3	3	2	4	2	5	3
Good Boys	2	3	3	7	4	6	3	3			
The Peanut Butter Falcon	3	3	3	5	3	3	8	3	3		
The Possession of Hannah Grace	5	4	2	4	5	2	4	3			
Rambo: Last Blood	4	3	4	8	5	4	3	6	7		

Table S2: Number of annotations selected from each movie clip as inliers for majority consensus ground truth generation out of a possible 10 per clip.

Movie Name	Movie Clips											Total
	C1	C2	C3	C4	C5	C6	C7	C8	C9	C10	C11	
The Hustle	7	8	3	11	1	7	2	4	3	1	4	51
Good Boys	5	6	19	11	6	6	4	4				61
The Peanut Butter Falcon	13	6	13	2	3	3	5	5	8			58
The Possession of Hannah Grace	6	9	9	3	8	6	3	7				51
Rambo: Last Blood	3	3	11	14	16	13	10	19	17			106

Table S3: Number of excerpts with constant violence extracted from each movie clip based on the number of constant segments observed in each movie's clips' fused trapezoidal segment sequence.

	Spearman correlations		
**Movie Name**	**Batch 1 vs. 1-2**	**Batches 1-2 vs. 1-3**	**Batches 1-3 vs. 1-4**
The Hustle	0.74	0.78	0.93
Good Boys	0.71	0.79	0.93
The Peanut Butter Falcon	0.80	0.90	0.81
The Possession of Hannah Grace	0.83	0.94	0.92
Rambo: Last Blood	0.84	0.89	0.95

Table S4: Rank-based measures of agreement between the candidate ground truth signals produced using the specified ranges of batches of pairwise comparisons. Each batch comprised 5000 unique movie clip violence comparisons.

Movie Name	Cut #	Kendall's τ	CCC	Agreement Metrics<br>ICC(1,k)	Krippendorff's α	SDA
The Hustle	1	.28 (.23)	.89 (.28)	.72 [.69, .75]	.03	.69 (.32)
	2	.01 (.00)	.67 (.47)	.97 [.96, .97]	-.48	.93 (.08)
	3	.64 (.25)	.79 (.11)	.87 [.85, .89]	.15	.93 (.05)
	4	.23 (.25)	.13 (.01)	.21 [.10, .31]	-.08	.28 (.12)
	5	.17 (.11)	.05 (.07)	-32.61 [-37.46, -28.28]	-.49	-.40 (.56)
	6	.41 (.09)	.23 (.31)	-1.99 [-2.46, -1.57]	-.08	.03 (.25)
	7	.65 (.00)	.24 (.00)	.37 [.26, .47]	.30	.80 (.00)
	8	.12 (.41)	.19 (.36)	.01 [-.11, .13]	-.06	.56 (.24)
	9	-.12 (.00)	.31 (.00)	.41 [.29, .51]	-.10	.17 (.00)
	10	—	1.00 (.00)	—	1.00	1.00 (.00)
	11	.27 (.24)	.12 (.17)	-.21 [-.71, .16]	-.09	.14 (.35)
Good Boys	1	.74 (.00)	.70 (.00)	.84 [.81, .86]	.35	.97 (.00)
	2	.35 (.08)	.18 (.10)	.28 [.18, .37]	.07	.17 (.34)
	3	.15 (.27)	.19 (.28)	-.65 [-.90, -.44]	-.11	-.09 (.18)
	4	.30 (.22)	.25 (.23)	.10 [-.01, .20]	.04	.59 (.20)
	5	.37 (.21)	.80 (.17)	.99 [.98, .99]	-.05	.91 (.06)
	6	.32 (.36)	.23 (.22)	.75 [.71, .78]	.04	.48 (.39)
	7	.73 (.19)	.59 (.29)	.70 [.65, .73]	.33	.98 (.02)
	8	.23 (.22)	.64 (.11)	.81 [.78, .83]	.03	.78 (.11)
The Peanut Butter Falcon	1	-.01 (.20)	.10 (.28)	.92 [.91, .93]	-.04	-.15 (.56)
	2	.35 (.10)	.33 (.14)	.55 [.49, .61]	-.07	.49 (.14)
	3	.65 (.27)	.46 (.27)	.48 [.41, .54]	.08	.55 (.32)
	4	.13 (.39)	.01 (.12)	-1.79 [-2.16, -1.45]	-.15	.46 (.33)
	5	.80 (.10)	.81 (.26)	.89 [.88, .91]	.21	.24 (.57)
	6	.38 (.08)	.12 (.10)	.43 [.34, .50]	.19	.81 (.07)
	7	.48 (.26)	.47 (.33)	.88 [.87, .89]	.02	.55 (.56)
	8	.62 (.19)	.16 (.10)	.30 [.19, .39]	.23	.52 (.25)
	9	-.05 (.39)	-.04 (.29)	-0.13 [-0.35, .06]	-.10	.27 (.08)
The Possession of Hannah Grace	1	.00 (.55)	.31 (.44)	.68 [.64, .72]	.00	.80 (.13)
	2	.22 (.21)	.49 (.38)	.36 [.27, .44]	-.02	.31 (.35)
	3	.47 (.00)	.31 (.00)	-1.40 [-1.81, -1.04]	.10	.67 (.00)
	4	.30 (.13)	.11 (.14)	-.39 [-.58, -.22]	-.11	.38 (.47)
	5	.39 (.16)	.46 (.36)	.87 [.85, .88]	.19	.57 (.18)
	6	.08 (.00)	.37 (.00)	.52 [.44, .58]	-.01	-.14 (.00)
	7	.16 (.09)	.02 (.04)	-7.24 [-8.42, -6.17]	-.32	.43 (.48)
	8	-.05 (.00)	.57 (.61)	-.67 [-.91, -.46]	.11	.40 (.51)
Rambo: Last Blood	1	.65 (.33)	.83 (.14)	.96 [.96, .97]	.06	.51 (.46)
	2	.64 (.11)	.66 (.04)	.90 [.89, .91]	.32	.95 (.02)
	3	.57 (.13)	.44 (.22)	.63 [.58, .67]	.22	.69 (.12)
	4	.61 (.14)	.66 (.19)	.92 [.91, .93]	.09	.41 (.18)
	5	.45 (.18)	.28 (.29)	.59 [.54, .64]	.02	.40 (.23)
	6	.51 (.26)	.32 (.13)	.56 [.50, .61]	.03	.50 (.42)
	7	.73 (.04)	.89 (.01)	.96 [.95, .97]	.11	.61 (.10)
	8	.52 (.18)	.51 (.21)	.83 [.81, .85]	.02	.16 (.39)
	9	.51 (.11)	.54 (.15)	.88 [.85, .90]	.06	.04 (.17)

Table S5: Various agreement metrics computed per movie clip for each movie among all selected, cleaned, and aligned inlier annotations. Entries with two values are formatted, "mean value (standard deviation)", while entries with three give the value and 95% confidence interval: "value [lower CI, upper CI]". A dash indicates the measure was not computable (NaN).
